# Biochemical characterization of recombinant influenza A polymerase heterotrimer complex: Endonuclease activity and evaluation of inhibitors

**DOI:** 10.1371/journal.pone.0181969

**Published:** 2017-08-15

**Authors:** Weimei Xing, Ona Barauskas, Thorsten Kirschberg, Anita Niedziela-Majka, Michael Clarke, Gabriel Birkus, Perry Weissburg, Xiaohong Liu, Brian E. Schultz, Roman Sakowicz, HyockJoo Kwon, Joy Y. Feng

**Affiliations:** Gilead Sciences, Inc., Foster City, California, United States of America; University of Geneva, SWITZERLAND

## Abstract

Influenza polymerase is a heterotrimer composed of polymerase acidic protein A (PA) and basic proteins 1 (PB1) and 2 (PB2). The endonuclease active site, located in the PA subunit, cleaves host mRNA to prime viral mRNA transcription, and is essential for viral replication. To date, the human influenza A endonuclease activity has only been studied on the truncated active-site containing N-terminal domain of PA (PA_N_) or full-length PA in the absence of PB1 or PB2. In this study, we characterized the endonuclease activity of recombinant proteins of influenza A/PR8 containing full length PA, PA/PB1 dimer, and PA/PB1/PB2 trimer, observing 8.3-, 265-, and 142-fold higher activity than PA_N_, respectively. Using the PA/PB1/PB2 trimer, we developed a robust endonuclease assay with a synthetic fluorogenic RNA substrate. The observed *K*_m_ (150 ± 11 nM) and *k*_*cat*_ [(1.4 ± 0.2) x 10^-3^s^-1^] values were consistent with previous reports using virion-derived replication complex. Two known influenza endonuclease phenylbutanoic acid inhibitors showed IC_50_ values of 10–20 nM, demonstrating the utility of this system for future high throughput screening.

## Introduction

Despite a highly successful vaccination program, influenza viruses infect 15–60 million people in the US each year, resulting in >200,000 hospitalizations and 3,000–49,000 deaths [1–3]. There is a significant unmet medical need for new influenza antivirals due to the increasing prevalence of resistance to the most commonly prescribed antiviral, oseltamivir, and widespread resistance to other existing agents such as amantadine and rimantadine [4]. Combination therapies with novel antiviral agents may be especially useful in controlling both viral infection and the emergence of drug resistance [5].

**Fig 1:**
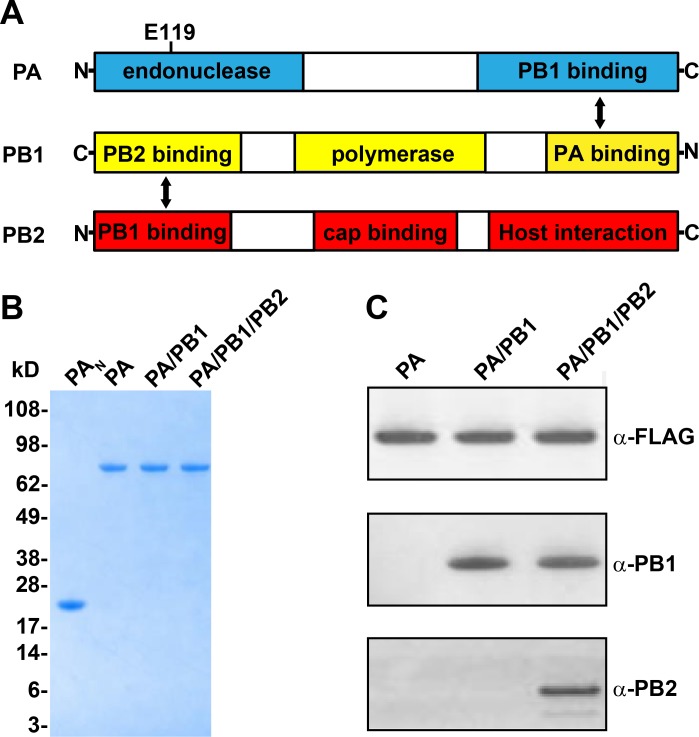
Biochemical characterization of purified influenza polymerase proteins. (A) Diagram showing the interactions between the PA, PB1, and PB2 subunits. (B) Coomassie staining of purified PA proteins. 1 μg of PA_N_ (lane 1), PA (lane 2), PA/PB1 dimer (lane 3) and PA/PB1/PB2 trimer (lane 4) were analyzed by Coomassie Blue-stained SDS-PAGE. Molecular weights of protein standards are indicated on the left. (C) Western blot detection of purified proteins. 100 ng of PA, PA/PB1 dimer, and PA/PB1/PB2 trimer were subjected to SDS-PAGE and transferred to PVDF membrane. The membrane was blotted with anti-FLAG, anti-PB1 and anti-PB2 antibodies for detection of PA, PB1 and PB2 proteins respectively.

The influenza virus belongs to the Orthomyxoviridae family and contains three types: type A, B, and C. As of today, the influenza A virus is the only type that causes both epidemics and pandemics[6]. Influenza A contains a genome with eight single-stranded, negative-sense RNA segments [4]. The virus can be classified into antigenic subtypes based on two different surface glycoproteins, hemagglutinin (HA) and neuraminidase (NA) [7]. Newly synthesized viral ribonucleoprotein (RNP) complexes are exported from the nucleus to the cytoplasm by the nuclear export protein (NEP) and the matrix protein M1 [4]. Each RNP is composed of one copy of viral RNA, one RNA-dependent RNA polymerase (RdRp), and multiple copies of viral nucleoprotein (NP). NPs encapsidate viral RNA protecting it from degradation while RdRP binds to the 3’ and 5’ends of viral RNA. Influenza A RdRp is a heterotrimer consisting of polymerase acidic protein (PA), which contains the endonuclease active site and a PB1 interaction domain; polymerase basic protein 1 (PB1), which contains the RdRp active site; and polymerase basic protein 2 (PB2) which is responsible for binding 7-methylguanylate capped RNA (Fig 1A). RdRp catalyzes two types of reactions: de novo synthesis of new viral RNA (RNA replication) and transcription of viral mRNA using a unique “cap-snatching” mechanism. For the latter, the PB2 subunit binds to a host 5’-capped pre-mRNA, followed by PA endonuclease-catalyzed cleavage at 10–13 nucleotides from the cap, to yield a primer that primes PB1-catalyzed viral mRNA transcription [7]. The cleavage of host mRNA and generation of primers for the transcription of the viral genome is critical for viral replication. Due to this unique mechanism of viral transcription and the low homology of PA with host proteins, the endonuclease is an attractive target for antiviral drug development.

The N-terminal domain of PA protein (PA_N_) has been crystallized and used for endonuclease inhibitor screening by many groups [7–10]. However, whether the truncated PA faithfully represents the biological function of the protein in vivo remains an important question. Several groups also reported the challenges they observed using PA_N_ in endonuclease activity assays. Yuan et. al. determined the crystal structure of PA_N_ containing the endonuclease active site motif. However, the high levels of endogenous nuclease in *E*. *coli* made it difficult to generate PA_N_ that was sufficiently pure for enzymatic studies [8]. Crepin et. al. studied metal ion binding properties and site-specific mutational analysis of endonuclease activity of PA_N,_ but without detailed enzymatic characterization of PA_N_ activity [11]. Thus, endonuclease inhibitor programs have faced significant challenges due to the use of PA_N_. Recently, a full-length form of PA was purified from insect cells; however, its weak binding affinity (*K*_d_ > 100 μM) towards the FRET-labeled RNA substrate precluded determination of steady state kinetic parameters (*k*_cat_ and *K*_m_) [12].

A number of specific influenza endonuclease inhibitors have been identified including diketoacid derivatives [13, 14] and recently reported heteroaryl bioisosteres with submicromolar potency [15–19]. Various methods have been developed to measure the inhibition of the endonuclease activity, such as radiometric methods using RNP from detergent disrupted viral particles and exogenous capped mRNAs [20, 21], and fluorescence polarization and Fluorescence Resonance Energy Transfer (FRET) based methods using both full length and PA_N_ and internally quenched nucleic acid probes [12, 13, 17, 22]. However, the lack of a highly active endonuclease prevents the development of a sensitive and high throughput screening platform.

In the current study, we expressed and purified full length PA, PA/PB1 dimer, and the PA/PB1/PB2 trimer. We showed that the endonuclease activity of the PA/PB1 dimer and PA/PB1/PB2 trimer is significantly higher than that of PA alone. We evaluated the endonuclease activity of these constructs in parallel using an optimized FRET-RNA substrate and developed an assay format suitable for high-throughput screening with sensitivity capable of measuring inhibitor potency in the nanomolar range.

## Materials and methods

### Materials and plasmids

**Fig 2:**
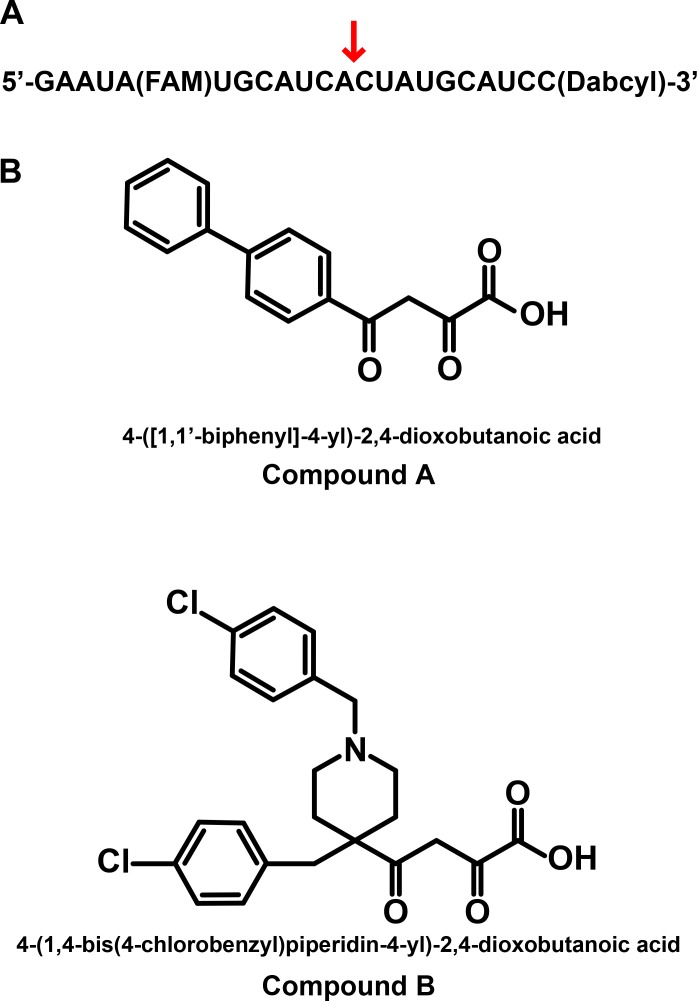
RNA substrate and known influenza endonuclease inhibitors used in this study. (A) Sequence of the RNA-FRET substrate with FAM fluorophore and Dabcyl quencher; (B) Structures and systematic names of compounds A and B tested in IC_50_ assays.

The PA, PB1, and PB2 genes of influenza A virus RNA polymerase were amplified by reverse-transcription and PCR from H1N1 strain A/PR/8. PA containing N-terminal 3x FLAG tag and PB1 were cloned into the BamHI and XhoI sites of pFastBac1 vector separately for baculovirus generation and expression of PA monomer and PA/PB1dimer. Baculovirus encoding the active site mutation E119A version of PA (PA-E119A) was generated by sited-directed mutagenesis (Agilent Technologies, Santa Clara, CA) of PA. For the expression of polymerase trimer, the full-length PB1 gene was cloned into the StuI and HindIII sites of pFastBac Dual vector (Life Technologies) under the PH promoter, resulting in pFastBac Dual–PB1. The full-length PB2 was then cloned into the KpnI and XhoI sites of pFastBac Dual–PB1 under the p10 promoter, resulting in pFastBac Dual containing PB1/PB2. Recombinant baculovirus was generated using Sf9 insect cells. The anti-PB1 and anti-PB2 antibodies were obtained from Santa Cruz Biotechology Inc. (Santa Cruz, CA). Anti-FLAG antibody was obtained from Sigma-Aldrich (St. Louis, MO). The RNA substrates used in the biochemical assays including the internally quenched fluorescent RNA substrate 5’-GAAUA(FAM)UGCAUCAUAGCAUCC(Dabcyl)-3’ (RNA-FRET) (Fig 2A) were purchased from TriLink Biotechnologies (San Diego, CA). Inhibitors (Compound A and B, Fig 2B) were synthesized by WuXi PharmaTech Inc. (Shanghai, China) and Gilead Sciences. All commercial reagents were of high purity (>95%).

### Protein expression and purification

For PA protein expression, the PA baculovirus was used to infect *Trichopulsia ni* (Hi5) cells at a density of 1.5×10^6^ cells/ml in ESF-921 medium (Expression Systems, Davis, CA). For PA/PB1 dimer expression, an optimized baculovirus ratio of PA and PB1 was used to co-infect Hi5 cells at 1.5×10^6^ cells/ml. For PA/PB1/PB2 trimer expression, an optimized baculovirus ratio of PA and PB1/PB2 was used to co-infect Hi5 cells. Infected cells were grown at 27°C, harvested 70 hours post infection by centrifugation and stored at -80°C.

To purify the PA protein, 1L cell pellet was suspended in 100 ml buffer A [25 mM HEPES (pH 7.6), 300 mM NaCl, 0.5% (v/v) Triton X100, and 0.5 mM TCEP] supplemented with two tablets of EDTA free Complete Protease Inhibitor Cocktail (Roche Diagnostics, Risch-Rotkreuz, Switzerland). Cells were lysed with a dounce homogenizer followed by sonication. The clarified supernatant containing PA protein was mixed with 4 ml of FLAG-M2 affinity resin (Sigma-Aldrich) equilibrated in Buffer A and incubated with rotation for 2 hours at 4°C. The resin was collected by centrifugation and washed with 40 ml of buffer B [25 mM HEPES (pH 7.6), 1 M NaCl, 5% Glycerol, 0.01% C12E8, and 0.5 mM TCEP], followed by 40 ml of buffer C [25 mM HEPES (pH 7.6), 300 mM NaCl, 0.01% C12E8, 5% glycerol, and 0.5 mM TCEP]. PA protein was eluted with buffer C supplemented with 200 ng/ml of FLAG peptide (iBIOSOURCE LLC, Foster City, CA). The eluted fractions were pooled, concentrated to 1 ml and further purified by size exclusion chromatography using a 24-ml Superdex 200 column (GE Healthcare, Little Chalfont, UK) equilibrated with buffer C. Fractions containing PA protein were concentrated and stored at -80°C for future use. The PA_N_ protein was purified as previously described [11]. Both PA/PB1 dimer and the PA/PB1/PB2 trimer were purified as described above for PA protein, except that after FLAG-affinity purification of PA/PB1/PB2, the protein was concentrated and further purified by size exclusion chromatography using a 24-ml Superose 6 column (GE Healthcare) equilibrated with buffer C followed by a 24-ml Superdex 200 column (GE Healthcare) equilibrated with buffer C. Fractions containing trimer were collected, concentrated and the presence of PA, PB1 and PB2 was verified by western blot. PA/PB1/PB2 trimer was stored at -80°C for future use. The active site E119A mutant forms of PA, PA/PB1 dimer, and PA/PB1PB2 trimer were expressed and purified similarly to their respective wild-type proteins.

### Mass spectrometry

Mass spectrometry of intact protein samples was performed with an Agilent 6210 Time of Flight Mass Spectrometer with a 4GHz upgrade and an Agilent 1200 Rapid Resolution HPLC. The samples were analyzed on an Agilent Zorbax 300 Extend C18 Rapid Resolution column at 70 °C, using reverse phase chromatography with a gradient from 20–90% acetonitrile containing 0.1% formic acid. Data were analyzed using Agilent Masshunter B.06 Qualitative Analysis with the Bioconfirm upgrade.

### Analytical ultracentrifugation

Sedimentation velocity experiments of PA, PA/PB1 dimer or PA/PB1/PB2 trimer were performed at 20°C in a ProteomeLab XL-A analytical ultracentrifuge (Beckman Coulter Inc., Fullerton, CA). Samples of 400 μl in Buffer C were loaded into a dual sector charcoal-filled Epon centerpiece. The samples were centrifuged at 42,000 rpm in an An50-Ti rotor, and sedimentation was monitored by absorbance at a wavelength of 280 nm. Data were analyzed with the program SEDFIT (NIH, Bethesda, MD), which generates a continuous c(s) distribution for the sedimenting species [23]. The program SEDNTERP [24] was used to estimate the partial specific volume of the proteins as well as the density and viscosity of the buffer solution.

### Size exclusion chromatography with multi-angle laser light scattering (SEC-MALS)

The molecular weights of PA, PA/PB1 dimer, and PA/PB1/PB2 trimer were analyzed by SEC-MALS using an Agilent Technologies 1200 LC HPLC system (Agilent Technologies, Santa Clara, CA) equipped with a Dawn Heleos 18-angle MALS light scattering detector, an Optilab rEX (refractometer) refractive index detector, a WyattQELS+ Quasi-Elastic-Light-Scattering detector, and ASTRA version 6 software (Wyatt Technology, Santa Barbara, CA, USA). A total of 100 μL (1 mg/ml) of PA, PA/PB1 dimer, or PA/PB1/PB2 trimer in buffer C was injected. The separation was conducted at ambient temperature, with the column eluent monitored at 280 nm at the mass detector. MALS analysis of the sample was performed continuously on the SEC column eluent as it passed through the MALS detector (fitted with a solid-state laser operating at 657 nm and a K5-flow cell). The QELS detector in the Heleos was at position 12 (100.3°) and was used to determine the hydrodynamic radii of the eluent peaks.

### Biochemical assays

To measure the endonuclease enzyme activity, dilutions of enzymes were prepared in a buffer containing 50 mM HEPES (pH 7.5), 100 mM KCl, 1 mM DTT, and either 5 mM MgCl_2_ or 1 mM MnCl_2_. Reactions were initiated with 1 μM RNA-FRET substrate at room temperature. Fluorescence was measured with excitation at 495 nm and emission at 516 nm at 30-second intervals for one hour on a Tecan Infinite M1000 plate reader (Tecan Group Ltd., Männedorf, Switzerland). Average reaction rates were calculated over the linear part of each progress curve using Magellan software (Tecan Group Ltd., Männedorf, Switzerland).

The *k*_cat_ and *K*_m_ values for the RNA-FRET substrate were measured in the presence of 10 nM influenza polymerase PA/PB1/PB2 trimer incubated at room temperature in a buffer containing 50 mM HEPES (pH 7.5), 100 mM KCl, 1 mM DTT, 1 mM MnCl_2_ and 5% (v/v) DMSO. All concentrations are final unless noted otherwise. Eight serial dilutions of the RNA-FRET substrate covering 10 nM -1 μM were prepared in buffer and added to initiate reactions. Fluorescence emission was monitored as described above. Average reaction rates (*v*) were calculated from the initial 30 minutes and used to calculate *K*_m_ using the Michaelis-Menten equation (Eq 1) where E_T_ = total enzyme concentration and S = substrate concentration, using GraphPad Prism 6 (GraphPad, La Jolla, CA). Total hydrolysis of the RNA-FRET substrate was achieved after a 10-min incubation with 10 U/mL RNAse I (New England Biolabs, Ipswitch, MA), and a standard curve was generated to calculate V_max_ and *k*_cat_.

$$v=\frac{E_{T}k_{cat}S}{K_{m}+S}$$ Eq 1

The inhibitory effects of small molecules were measured in the presence of 5 nM influenza polymerase PA/PB1/PB2 trimer pre-incubated for 15 minutes with various concentrations of inhibitors at room temperature in a buffer containing 50 mM HEPES (pH 7.5), 100 mM KCl, 1 mM DTT, 1 mM MnCl_2_, and 2% (v/v) DMSO. Reactions were initiated by addition of 200 nM RNA-FRET substrate and monitored as described above. Reaction rates were calculated by fitting the progress curve over the initial 30 minutes with a linear equation. Rates were plotted in GraphPad Prism 6 and fitted with a four- parameter dose response with variable slope equation to calculate half maximal inhibitory concentration (IC_50_) (Eq 2) where *x* = compound concentration, *Y* = % reaction rate as that of the DMSO control, and *s* = Hillslope.

$$Y=\frac{100}{1+{\left(\frac{x}{IC50}\right)}^{s}}$$ Eq 2

## Results

### Purification and characterization of influenza polymerase proteins PA, PA/PB1 dimer, and PA/PB1/PB2 trimer

Our protein expression and purification strategies involved co-expression of affinity tagged PA and untagged PB1 and PB2 subunits using baculovirus vector system in Hi5 cells, and purification of single or complex proteins employing FLAG-affinity and size exclusion chromatography (SEC). The full-length PA was >90% pure as judged by SDS-PAGE (Fig 1B), and its identity was confirmed by western blot using an anti-FLAG antibody (Fig 1C) and mass spectrometry (data not shown). The PA/PB1 dimer and PA/PB1/PB2 trimer were both purified to homogeneity with >90% purity. Since all three of the PA, PB1, and PB2 proteins have nearly identical molecular weights and migrated at the same location on SDS-PAGE gels (Fig 1B), the identities of each were confirmed by Western blot with anti-FLAG, anti-PB1 and anti-PB2 antibodies (Fig 1C). The integrity of these three proteins was also confirmed with their intact molecular weights by mass spectrometry analysis (data not shown). The active site mutant versions of PA (PA-E119A), PA/PB1 dimer (PA-E119A/PB1) and PA/PB1/PB2 (PA-E119A/PB1/PB2) were all purified using similar strategies. The mutant proteins were all >90% purity (SDS-PAGE gel) and their integrity were confirmed with their intact molecular weights by mass spectrometry analysis (data not shown).

**Fig 3:**
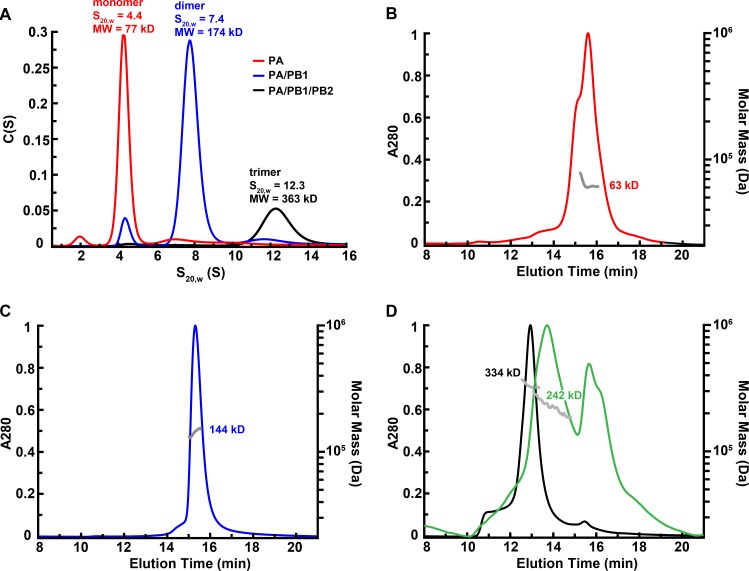
Biophysical characterization of purified influenza polymerase proteins. (A) Analytical ultracentrifugation analysis of purified proteins. For PA, PA/PB1 dimer and PA/PB1/PB2 trimer, analytical ultracentrifugation was performed in a buffer containing 25 mM HEPES (pH 7.6), 300 mM NaCl, 5% glycerol, 0.5 mM TCEP, and 0.01% C12E8. Sedimentation velocity analysis was performed at 42,000 rpm. Representative traces for each protein are shown. (B-D) SEC-MALS analysis of purified PA (B) PA/PB1 (C), and PA/PB1/PB2 in the presence (black) or absence (green) of detergent during SEC purification (D). The normalized UV absorbance trace (280 nm) is plotted on the left axis. The horizontal gray lines correspond to the calculated mass by SEC-MALS (right axis).

We further analyzed the oligomeric state of PA using analytical ultracentrifugation (AUC) and SEC-MALS. Based on the sedimentation velocity, PA exists primarily as a monomer of 77 kD (77%), which is in agreement with the calculated molecular weight (85 kD). Small amounts of dimer (10%) and trace amount of smaller species were also detected (Fig 3A), SEC/MALS showed PA with molecular mass of 63 kD (Fig 3B). The purified PA/PB1 dimer and PA/PB1/PB2 trimer were also characterized using AUC and SEC-MALS. According to sedimentation velocity measurements (Fig 3A), the PA/PB1 exists primarily as a heterodimer of 174 kD (81%, calculated molecular weight 172 kD) with a small percentage of tetramer (7%), high order oligomers (6%) and a monomer species (6%) which can be attributed to excess PA. The results of SEC/MALS for PA/PB1 dimer (Fig 3C) showed the molecular mass of 144 kD (90%) and higher order oligomeric species (10%). In AUC experiment, the PA/PB1/PB2 trimer sedimented primarily as a species with an apparent molecular weight of 363 kD (68%), and a small percentage of dimers or monomer (2% or 5%) and high order oligomers (26%) (Fig 3A). SEC/MALS analysis showed a major species (84%) with an apparent molecular weight of 334 kD and 11% oligomeric species (Fig 3D). The observed molecular weight for the polymerase heterotrimer in both methods is higher than calculated. When the affinity purified polymerase was subjected to SEC purification in the buffer containing no C12E8, the SEC-MALS analysis showed the major peak shifted to 242 kD, matching the calculated molecular weight of 258 kD for the heterotrimer, indicating that the 334 kD peak may be due to the presence of the bound detergent (Fig 3D).

### Evaluation of RNA-FRET substrate and metal-dependence

**Fig 4:**
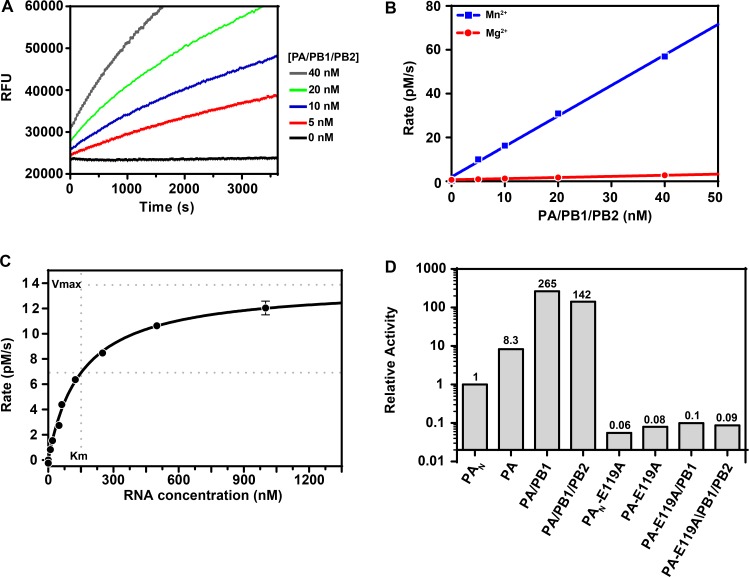
Enzymatic characterization of purified proteins. (A) The endonuclease activity of PA/PB1/PB2 trimer as a function of time and increasing protein concentration. Dilutions of the trimer were prepared in buffer containing 50 mM HEPES (pH 7.5), 100 mM KCl, 1 mM DTT, and 1 mM MnCl_2_. Reactions were initiated with 1 μM RNA-FRET substrate at room temperature and fluorescence was measured with excitation at 495 nm and emission at 516 nm at 30 second intervals. (B) Different endonuclease activity of the PA/PB1/PB2 trimer in the presence of manganese or magnesium. Dilutions of the trimer were prepared and assayed as described previously using either 1 mM MnCl_2_ or 5 mM MgCl_2_. The average reaction rates were calculated from the linear portion of each curve over a 30-minute reaction time and plotted as a function of the trimer enzyme concentration. (C) Determination of *k*_cat_ for the endonuclease activity of the PA/PB1/PB2 trimer and binding affinity (*K*_m_) for the RNA-FRET substrate. 10 nM PA/PB1/Pb2 was incubated in a buffer containing 1 mM MnCl_2_ as described previously. Two-fold serial dilutions of the RNA-FRET probe from 1 μM were prepared in buffer containing DMSO to 5% final reaction volume. Reactions were initiated with RNA dilutions at room temperature and fluorescence emission was monitored with excitation 495 nm and emission at 516 nm at 30 second intervals. Average rates were calculated from the linear portion of each curve and plotted as a function of RNA concentration. Total hydrolysis of the substrate was achieved by incubation for 10 minutes with 10 U/mL RNAse I and used to generate a standard curve used in the calculation of *k*_cat_. The *K*_m_ and *k*_cat_ values obtained were 150 ± 11 nM and (1.4 ± 0.2) x 10^-3^s^-1^, respectively.

The RNA-FRET substrate was designed using an optimized RNA sequence [25] and a FRET donor-quencher pair that yielded high signals and low background. The RNA showed saturable binding to the PA/PB1/PB2 trimer with *K*_m_ of 160 nM and the initial reaction rate increased proportionally as a function of enzyme concentration (Fig 4A). Based on the above observation, 5 nM trimer was chosen as the optimal condition for a 30-minute kinetic assay. The effect of the divalent cations Mg^2+^ and Mn^2+^ was also tested using the PA/PB1/PB2 trimer. The endonuclease activity was 27-fold higher in the presence of Mn^2+^ than Mg^2+^ (Fig 4B), consistent with previous reports [11, 12]. Due to the increased activity, manganese was used as the catalytic metal in following assays.

The cleavage products of the RNA-FRET substrates were visualized by polyacrylamide gel electrophoresis. As shown in S1 Fig, the Mn^2+^-catalyzed reaction formed significantly more cleavage products than Mg^2+^-catalyzed reactions. In the presence of Mn^2+^, two major cleavage products were formed, the first migrated between the 9–12 nt size markers which is consistent with the expected 11 nt cleavage product; the second major product migrated at 7 nt size marker. In addition, the substrate-dependence of the endonuclease activity of PA/PB1/PB2 trimer was studied using a 5’-m_7_G^1^-capped and uncapped RNA-FRET substrates. As shown in S2 Fig, the two substrates showed similar binding affinity but significantly different maximum reaction rates (*V*_*max*_). We selected the uncapped substrate due to its high signal/noise ratio and more suitable for high throughput assays.

### Enzymatic activity of PA, PA/PB1, and PA/PB1/PB2

The PA/PB1/PB2 trimer was characterized for its binding affinity to the RNA-FRET substrate (*K*_m_) and the endonuclease catalytic rate constant (*k*_*cat*_). The *K*_m_ and *k*_cat_ values were 150 ± 11 nM and (1.4 ± 0.2) × 10^-3^s^-1^, respectively (Fig 4C). These are in agreement with *K*_d_ of 10–170 nM and *k*_cat_ of (0.5–0.8) × 10^-3^s^-1^ from previous reports using viral RNP and capped RNA oligonucleotides [26, 27].

The endonuclease activity of purified full length PA, PA/PB1 dimer, and PA/PB1/PB2 heterotrimer were compared to PA_N_, the truncated endonuclease domain of PA. The endonuclease activity was measured by mixing various concentrations of PA, PA/PB1, or PA/PB1/PB2 with a fixed concentration of the internally quenched RNA-FRET substrate (1 μM) and the initial rates were calculated. Compared to the activity of PA_N_, full length PA, PA/PB1 dimer, and PA/PB1/PB2 trimer showed 8.3-, 265-, and 142-fold increases in activity, respectively (Fig 4D). Additionally, the active site mutation E119A led to complete loss of endonuclease activity in PA_N_, PA, the PA/PB1 dimer, and the PA/PB1/PB2 trimer, confirming that substrate cleavage by the corresponding wild-type enzymes was not due to any contaminating nuclease [11].

### Inhibition of endonuclease activity

**Fig 5:**
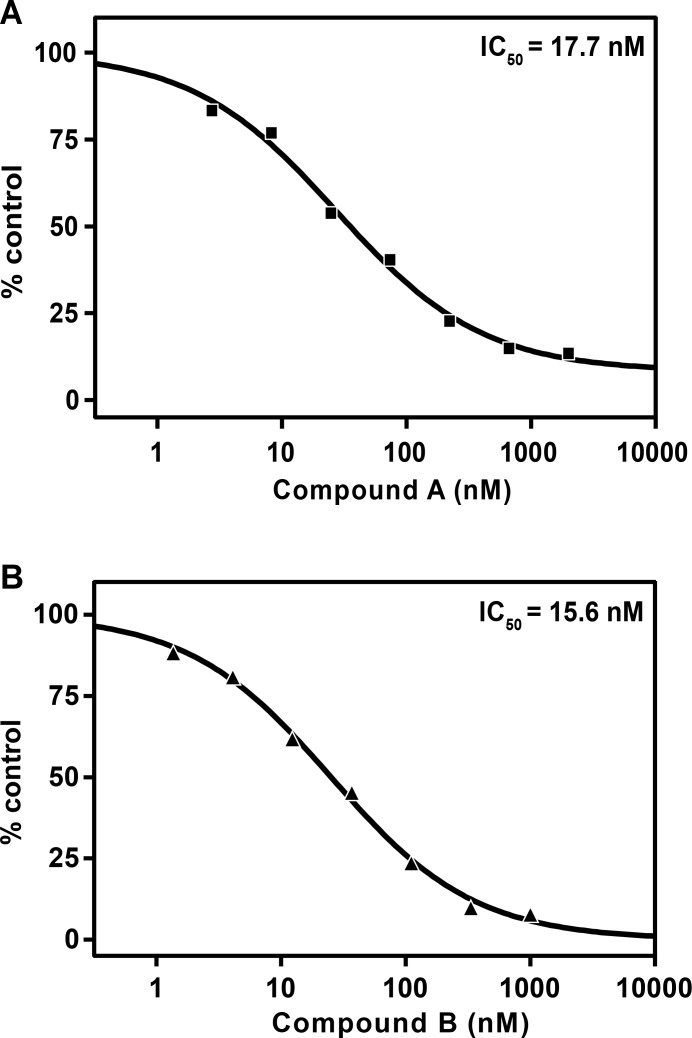
Inhibition of PA/PB1/PB2 trimer activity by influenza A endonuclease inhibitors. 5 nM trimer was pre-incubated for 15 minutes with three fold serial dilutions of compound A (A) or compound B (B) from 1000–2000 nM at room temperature in a buffer containing 50 mM HEPES (pH 7.5), 100 mM KCl, 1 mM DTT, 1 mM MnCl_2_, and DMSO (2% v/v). Reactions were initiated by addition of 200 nM RNA-FRET substrate and monitored as described above. Average reaction rates were calculated over the 30-minute reaction time. IC_50_ curves were fitted with a four parameter dose response with variable slope equation (GraphPad Prism 6).

The half maximal inhibitory concentrations (IC_50_) were determined for two reported influenza endonuclease inhibitors, 4-([1,1'-biphenyl]-4-yl)-2,4-dioxobutanoic acid (compound A), and 4-(1,4-bis(4-chlorobenzyl)piperidin-4-yl)-2,4-dioxobutanoic acid (compound B) [13, 14] (Fig 2B). Compound A and B showed potent inhibition of the trimer with IC_50_ values of 17.7 nM and 15.6 nM, respectively (Fig 5). Similar IC_50_ values were obtained against PA and PA/PB1 dimer for both compounds (data not shown).

## Discussion

Recently, the expression and purification of influenza polymerase trimer complex has been reported by a number of groups. High resolution X-ray crystal structures for influenza polymerase trimer complex have been solved for bat influenza A [28], human influenza B [29, 30] and influenza C viruses [31], providing unprecedented insights into the molecular mechanisms of the action of the polymerase. These groups applied two main strategies: (1) the polyprotein strategy by expressing PA-PB1-PB2 in tandem linked by Tobacco Etch Virus (Tev) protease cleavage site and later cleaved by Tev during purification to obtain the trimer complex [28–30]; and (2) the MultiBac system with one single baculovirus containing all of the three proteins for expression [31]. Swale et.al evaluated the expression of human influenza A polymerase complex using polyprotein strategy and identified PB2 as the bottleneck limiting the level of the expression [32]. In our work, we expressed the recombinant human influenza A polymerase complex by cloning PB1 and PB2 in the dual promoter vector in one single baculovirus and cloning N-terminal FLAG tagged PA in the second baculovirus, and optimized the baculovirus ratio to obtain the expression of the complex. We observed that tag-free PB1 and PB2 were important for successful expression and purification of active human influenza A polymerase complex. All of the three forms of the purified proteins (full length PA, PA/PB1 dimer, and PA/PB1/PB2 trimer) were confirmed to be intact, of high purity, and predominately monodisperse (>70%) by various analysis methods including Western blot, mass spectrometry, analytical ultracentrifugation, and size exclusion chromatography with multi-angle laser light scattering. To our knowledge, this is the first report providing side-by-side comparison of the endonuclease activity of three different forms of the functional PA protein. Compared to the widely used truncated form of PA, PA_N_ [7–9, 11], the full length PA showed a significant 8.3-fold increase in endonuclease activity (Fig 4D). Furthermore, the PA/PB1 dimer and the PA/PB1/PB2 trimer demonstrated 32-fold and 17-fold higher endonuclease activity than full-length PA, respectively (Fig 4D). This PB1- and PB2-enhanced endonuclease activity is likely resulted from conformational changes or structural modulation. The aggregation onset temperature measured by static light scattering (SLS) for PA (42.4°C) was higher than PA/PB1 (38.7°C) and PA/PB1/PB2 (36.2°C) (S3 Fig), indicating that PA adopted a different conformation when in isolation than when in complex with PB1 and PB2, rather than increased stability of multi-protein complex. This is in agreement with published crystallography studies of the influenza polymerase heterotrimer demonstrating interactions between PA, PB1, and PB2 subunits as well as conformational changes upon vRNA binding [28–31, 33].

Whether Mn^2+^ or Mg^2+^ is the preferred metal ion for the influenza endonuclease has been controversial. Conclusions from early studies using virion-derived RNP varied from absolute need for Mn^2+^ to a modest 2-fold activity increase by Mn^2+^ over Mg^2+^ [34, 35]. Recent X-ray crystallographic structure and activity studies on PA_N_ or full length PA did not clarify this issue, suggesting either Mn^2+^ [7, 9, 11, 12] or Mg^2+^ [8, 36] as the more relevant divalent ion. Our study demonstrates that the endonuclease activity of PA/PB1/PB2 trimer was 27-fold higher with Mn^2+^ than with Mg^2+^ (Fig 4B). This is consistent with the observed 500-fold higher binding affinity of PA_N_ towards Mn^2+^ than Mg^2+^ using isothermal titration calorimetry measurements by Crepin et al. [11], suggesting Mn^2+^ as the more relevant divalent ion for the endonuclease activity.

In this study, we have described a robust and reproducible assay using 5 nM influenza polymerase PA/PB1/PB2 trimer and 200 nM RNA substrate ([S] ~ *K*_m_), which is suitable for both high-throughput screening and characterization of potent inhibitory compounds in lead optimization. This offers an improvement over previously reported assays utilizing full length PA or PA_N_ in the 0.8–11 μM range [12, 13, 17, 22, 37]. We demonstrated that two known 4-substituted diketoacid inhibitors were potent endonucleases inhibitors with ~ 20 nM IC_50_ values. This value is significantly lower than the originally reported IC_50_ of 3.7 μM for Compound A using endonuclease from detergent disrupted viral particles [14], or 1.1 μM for Compound B in a cell-based in vitro transcription assay [13]. In our hands, the FRET-based endonuclease assay rank-ordered compound potency in agreement with traditional radiometric assays but offers a format amenable to high throughput screening. In summary, we have successfully produced a full length PA and stoichiometric PA/PB1 dimer and PA/PB1/PB2 trimer with high endonuclease activity. The dramatically increased endonuclease activity in these reconstituted complexes should be useful for high throughput drug discovery of future influenza antiviral agents.

## Supporting information

S1 Fig PA/PB1 dimer catalyzed cleavage of RNA substrate. (PDF)

S2 Fig Endonuclease cleavage of uncapped (A) and capped (B) RNA-FRET substrates.
(PDF)

S3 Fig Onset of thermal aggregation for PA, PA/PB1 and PA/PB1/PB2. (PDF)
