# Supplementary material for: Biochemical characterization of recombinant influenza A polymerase heterotrimer complex: Endonuclease activity and evaluation of inhibitors
Source: PLoS One. 2017 Aug 15;12(8):e0181969. doi: 10.1371/journal.pone.0181969 (PMC5557545; doi:10.1371/journal.pone.0181969)
Supplement: S1 Fig — (PDF) [file pone.0181969.s001.pdf]

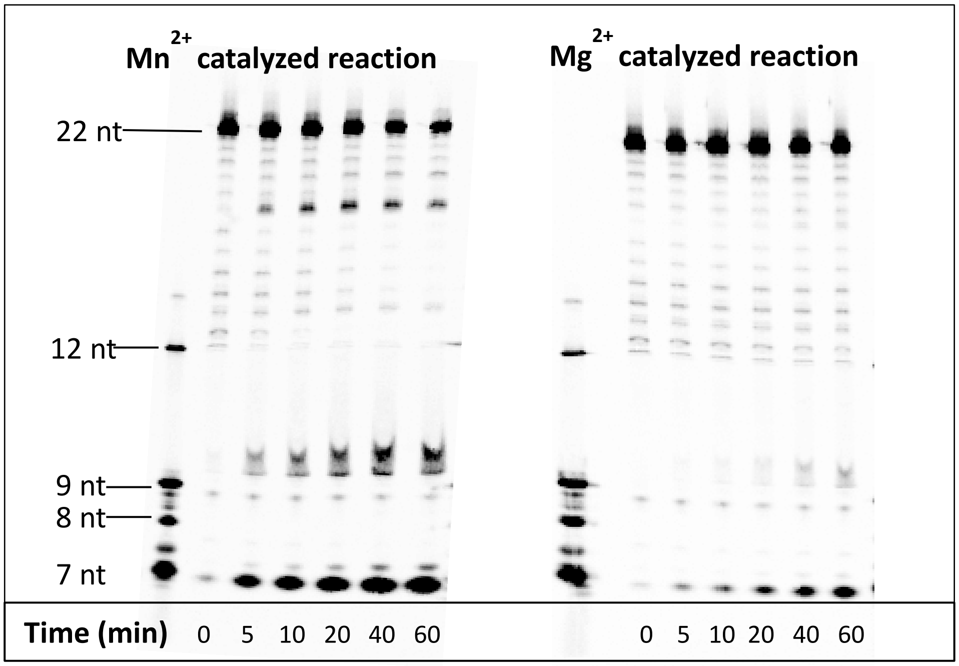

S1 Fig. PA/PB1 dimer catalyzed cleavage of RNA substrate. RNA-FRET substrate and oligonucleotide size markers were 5’ end labeled with γ-^{33}P-ATP (PerkinElmer, Waltham, MA) using T-4 polynucleotide kinase (New England Biolabs, Ipswich, MA) according to manufacturer’s instructions. 1 μM RNA substrate was incubated with 640 nM PA/PB1 dimer at room temperature in buffer containing 50 mM HEPES pH 7.5, 100 mM KCl, 1 mM DTT and 1 mM $MnCl_2$ or 5 mM $MgCl_2$. At time zero, 5, 10, 20, 40 and 60 minutes, 10 μL of the reactions were quenched with 100 mM EDTA in 90% formamide loading dye containing trace bromphenol blue and xylene cyanol. Quenched reactions were heated at 90 °C for 5 minutes, then products were separated by 20% polyacrylamide gel electrophoresis and visualized by autoradiography.
