# Supplementary material for: Biochemical characterization of recombinant influenza A polymerase heterotrimer complex: Endonuclease activity and evaluation of inhibitors
Source: PLoS One. 2017 Aug 15;12(8):e0181969. doi: 10.1371/journal.pone.0181969 (PMC5557545; doi:10.1371/journal.pone.0181969)
Supplement: S2 Fig — Endonuclease cleavage of uncapped (A) and capped (B) RNA-FRET substrates. (PDF) [file pone.0181969.s002.pdf]

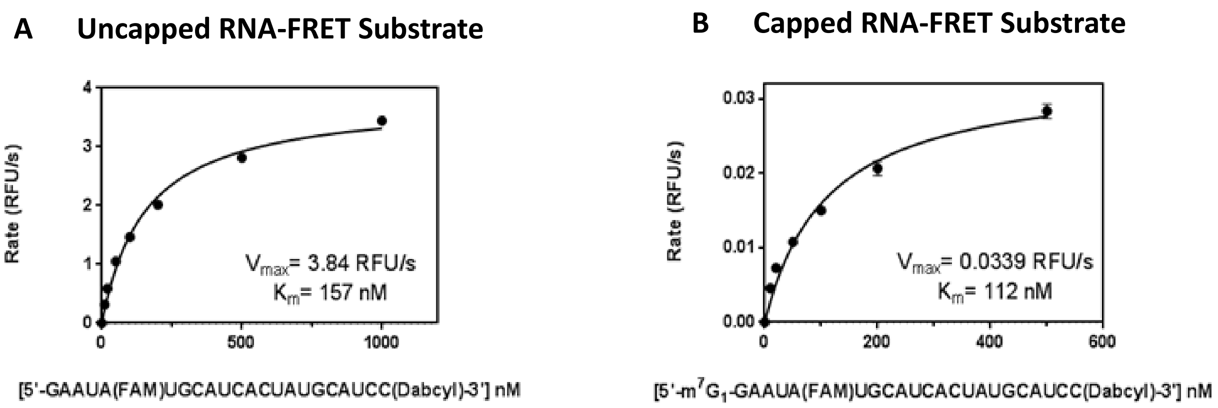

S2 Fig. Endonuclease cleavage of uncapped (A) and capped (B) RNA-FRET substrates. m_7G^1 capped and uncapped fluorogenic RNA substrates were custom synthesized by TriLink Biotechnologies (San Diego, CA). Dilutions of substrate were incubated with 8.2 nM trimer in buffer containing 50 mM HEPES (pH 7.5), 100 mM KCl, 1 mM DTT, and 1 mM $MnCl_2$. Fluorescence was monitored continuously over 1 hr with excitation at 494 nm and emission at 520 nm. Average reaction rates were fit with GraphPad Prism with a Michaelis-Menten equation to calculate V_{max} and K_m.
