# Supplementary material for: Biochemical characterization of recombinant influenza A polymerase heterotrimer complex: Endonuclease activity and evaluation of inhibitors
Source: PLoS One. 2017 Aug 15;12(8):e0181969. doi: 10.1371/journal.pone.0181969 (PMC5557545; doi:10.1371/journal.pone.0181969)
Supplement: S3 Fig — (PDF) [file pone.0181969.s003.pdf]

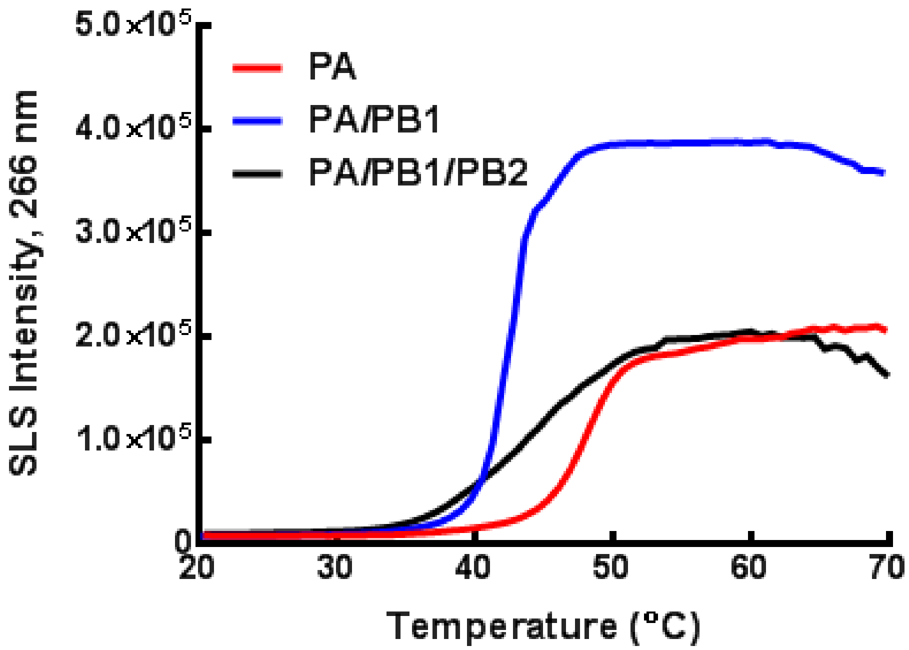

S3 Fig. Onset of thermal aggregation for PA, PA/PB1 and PA/PB1/PB2. PA, PA/PB1 and PA/PB1/PB2 was in buffer containing 25 mM HEPES (pH 7.6), 300 mM NaCl, 5% glycerol, 0.5 mM TCEP, and 0.01% C12E8, the concentrations for each protein was 0.5 mg/ml, 1.0 mg/ml, and 0.4 mg/ml, respectively. Aggregation onset of the proteins were determined by measuring the increase in static light scattering (SLS) intensity at 266 nm over the thermal ramp from 20 ^{o}C to 70 ^{o}C using UNcle instrument (Unchained Labs, Pleasanton, CA). Each protein was analyzed in triplicate and the average T_{agg} values were shown.
